# Identification of New Therapeutic Targets for Gastric Cancer With Bioinformatics

**DOI:** 10.3389/fgene.2020.00865

**Published:** 2020-08-18

**Authors:** Yang Li, Jin-Shen Wang, Tao Zhang, Hong-Chang Wang, Le-Ping Li

**Affiliations:** ^1^Department of Gastrointestinal Surgery, Shandong Provincial Hospital, Cheeloo College of Medicine, Shandong University, Jinan, China; ^2^Department of Biostatistics, School of Public Health, Shandong University, Jinan, China

**Keywords:** gastric cancer, prognosis, mutation, CNA, purity, TMB, TME score

## Abstract

We aimed to identify new targets affecting gastric cancer (GC) prognosis. Six target genes were identified from hub genes based on their relationship with important factors affecting tumor progression, like immune infiltration, purity, tumor mutation burden (TMB), and tumor microenvironment (TME) score. The effect of target genes’ somatic mutations and copy number alteration (CNA) was examined to determine their effect on GC prognosis. Six target genes (*FBN1*, *FN1, HGF, MMP9, THBS1*, and *VCAN*) were identified. Reduced expression of each target gene, except *MMP9*, indicated better prognosis and lower grade in GC. *FBN1, THBS1*, and *VCAN* showed lower expression in stage I GC. Non-silencing mutations of the six genes played a role in significantly higher TMB and TME scores. *THBS1* mutation was associated with earlier stage GC, and *VCAN* mutation was associated with lower grade GC. However, patients with target gene CNA displayed higher tumor purity. *MMP9*, *THBS1*, and *VCAN* CNA was associated with lower grade GC, while *FBN1* CNA reflected earlier T stage. Additionally, the target genes may affect GC prognosis by influencing multiple oncogenic signaling pathways. *FBN1, FN1, HGF, MMP9, THBS1*, and *VCAN* may be new GC prognostic targets by affecting tumor purity, TMB, TME score, and multiple oncogenic signaling pathways.

## Introduction

Gastric cancer (GC) is a disease with a high incidence and high mortality around the world (Kamangar et al., 2006). With the development of medical technology, treatment of GC with surgery combined with chemoradiotherapy is gradually becoming more effective, but the 5-year survival rate remains subpar (Ferlay et al., 2015; Li Y. et al., 2019). In particular, post-operative chemotherapy has a major impact on the prognosis and survival of GC patients (Kunkler, 1994; van de Velde, 2008; Bernini and Bencini, 2012). In recent years, a variety of regimens have been studied to identify an improved therapeutic approach. However, treatment can vary greatly in patients with the same pathological type and stage, or even in those with similar expression of hub genes (Yan et al., 2018; Huang et al., 2019). Therefore, it is important to identify new targets influencing the prognosis of GC.

Immune infiltration, tumor purity, tumor mutation burden (TMB), and tumor microenvironment (TME) score have been investigated as important factors affecting tumor prognosis and chemoradiotherapy (Yoshihara et al., 2013; Aran et al., 2015; Hellmann et al., 2018; Lazar et al., 2018; Zeng et al., 2018; Pan et al., 2019). Higher TMB, TME score, and tumor purity indicate a better prognosis. Somatic mutations and copy number alterations (CNAs) of genes are also key factors affecting tumor development through complex mechanisms (Cutcutache et al., 2016; Liang et al., 2016; Li et al., 2018; Li X. et al., 2019). Large-scale studies have aimed to explore the molecular changes in GC that may reveal new and important targets in its therapy.

**FIGURE 1:**
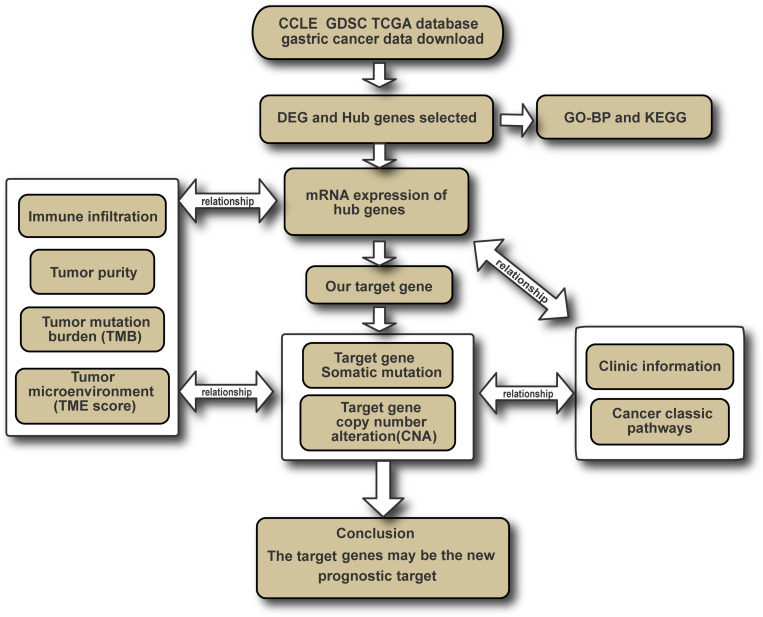
The analysis chart of our study.

In our study, we analyzed the Cancer Cell Line Encyclopedia (CCLE), The Cancer Genome Atlas (TCGA), and the Genomics of Drug Sensitivity in Cancer (GDSC) database, to select six genes (*FBN1, FN1, HGF, MMP9, THBS1*, and *VCAN*) as target genes. Based on their expression, mutation, and CNA, along with analysis of their relationship with clinical information, TMB, TME score, tumor purity, immune infiltration, and classic oncogenic signaling pathways, we thought that these genes could serve as new therapeutic and prognostic targets (Figure 1).

## Materials and Methods

### Data Collection and Hub Gene Selection

RNA-seq data from gastric cells was downloaded from the CCLE database^1^. 5-FU and oxaliplatin drug sensitivity for different gastric cancer cell lines were obtained from the GDSC database^2^. Primary somatic mutation, RNA-seq, and clinical data from TCGA Stomach Cancer (STAD) was downloaded from TCGA^3^. GEPIA2^4^ was used for the comparison of gene expression between STAD and normal tissue from GTEx dataset. MC3 gene-level non-silent mutation data and gistic2 threshold of copy number data was obtained from UCSC Xena website^5^. The EdgeR package was used to analyze differential gene expression (*P* < 0.05, | log FC| ≥ 1). Gene Ontology (GO) and Kyoto Encyclopedia of Genes and Genomes (KEGG) were analyzed using DAVID^6^. The protein interaction network was constructed in String^7^. Using 12 algorithms and a combination of the most commonly used methods (Degree and MCC) referenced elsewhere (Chin et al., 2014; Wang W. et al., 2019), we selected the top 30 hub genes using cytohubba in Cytoscape (version 3.7.1).

### Calculation of Immune Infiltration, Tumor Purity, TMB, and TME Score

The immune cells portion was calculated using the CIBERSORTS^8^ method and the LM22 gene signature were used for immune infiltration analysis (Chen et al., 2018). All results were considered statistically significant if *P* < 0.05. Correlation between immune cells and hub genes was calculated by Pearson (| R| > 0.4 and *P* < 0.05). TMB was calculated based on somatic mutation data. TME score was calculated as previously described (Zeng et al., 2019). Tumor purity, immune score, and stromal score were calculated using the ESTIMATE package (Yoshihara et al., 2013). The best cut-off values of genes for Kaplan-Meier were calculated by survminer package through R software (Li M. et al., 2019). Ten classic oncogenic signaling pathways and important genes among them were from Sanchez-Vega et al. (2018).

### Statistical Analysis

Statistical analyses were conducted using R software (3.6.1 version) and SPSS version 23.0. Graphical representations were generated using GraphPad Prism 8. Mann-Whitney test and Kruskal-Wallis tests were used for continuous variables with two or more groups, respectively. Chi squared was used for categorical variables. The Pearson coefficient was used to test for correlations.

## Results

### Differential Gene Expression and Hub Genes

**FIGURE 2:**
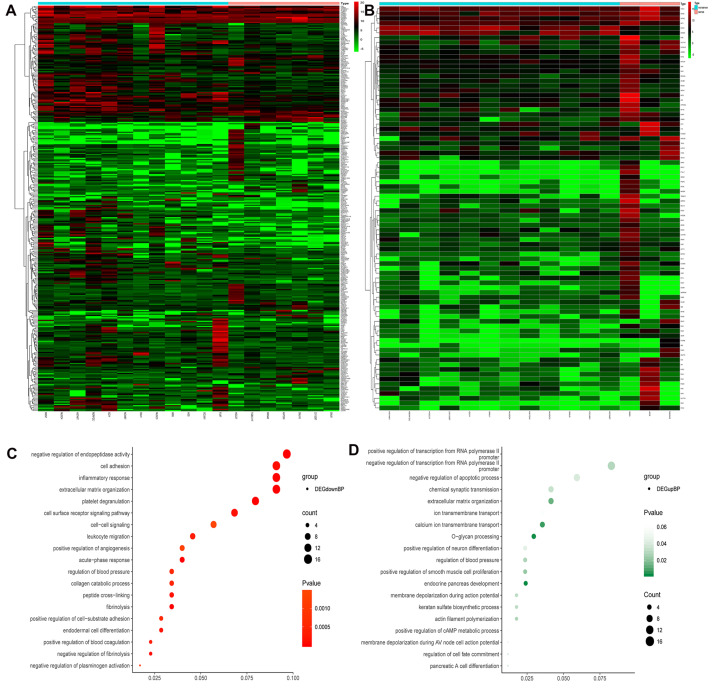
Heat maps and GO-BP of differential gene expression analysis in sensitized and drug-resistant cells. **(A)** Heat map of the genes differentially expressed between 5FU-sensitive and non-sensitive cells, **(B)** heat map of differentially expressed genes in Oxaliplatin groups, **(C)** GO analysis of downregulated genes revealed that these genes were primarily involved in the negative regulation of endopeptidase activity, cell adhesion, inflammatory response, extracellular matrix organization, etc., **(D)** upregulated genes were primarily involved in positive and negative regulation of transcription from RNA polymerase II promoter, negative regulation of apoptosis, chemical synaptic transmission, etc.

From the GDSC database, genes were analyzed for differences in expression between sensitive and drug-resistant cells of 5-Fluorouraci (5-FU) and Oxaliplatin, respectively. Comparing sensitive and drug-resistant cells revealed 328 and 84 differentially expressed genes, respectively (16 duplicate genes) (Figures 2A,B and Supplementary Table S1). Gene Ontology Biological process (GO-BP) analysis showed that the differentially expressed genes were primarily involved in negative regulation of endopeptidase activity, cell adhesion, inflammatory response, and O-glycan processing (Figures 2C,D). These biological functions are closely related to the development and prognosis of GC (Duarte et al., 2016; Cui et al., 2017; Mukai et al., 2017; Geng et al., 2018).

### Selection of Hub Genes

**FIGURE 3:**
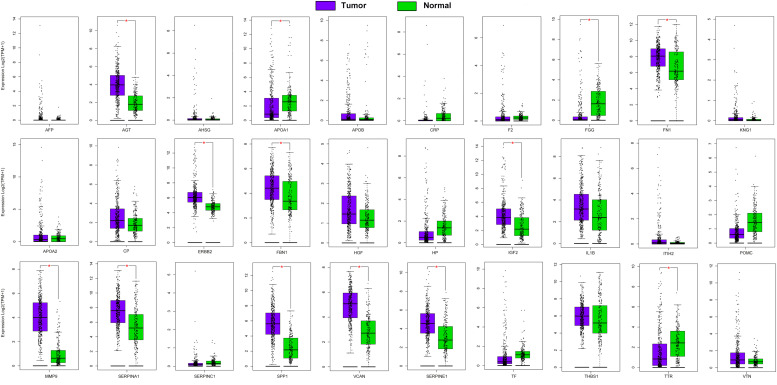
Expression of 30 hub genes in TCGA-STAD and normal tissue from GTEx dataset. *P < 0.05.

All differentially expressed genes were used in the String website to construct a protein interaction network, and key hub genes were identified by cytohubba in Cytoscape. Thirty hub genes were selected using 12 algorithms (Supplementary Table S2) and combined the most frequent methods (Degree and MCC) referenced in other studies. The expression levels of the 30 hub genes in TCGA Stomach adenocarcinoma (TCGA-STAD) and normal tissue from GTEx dataset were got from GEPIA2 (Figure 3). It showed that 10 genes (AGT, FN1, ERBB2, FBN1, IGF2, MMP9, SERPINA1, SPP1, VCAN and SERPINE1) were significantly higher expressed in tumor tissues, while 3 genes including APOA1, FGG, and TTR, were significantly lower expressed in tumor tissues.

### Correlation Between Hub Genes and Immune Infiltration

**FIGURE 4:**
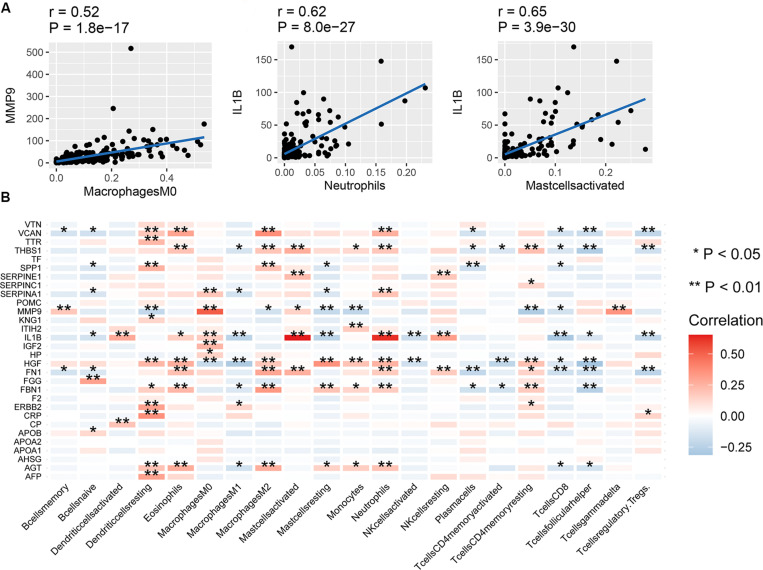
Correlation between hub genes and immune infiltration. **(A)** Correlation between MMP9 and macrophages (M0), IL1B and neutrophils, and IL1B and activated mast cells, **(B)** correlation between the expression of the 30 hub genes and 22 types of immune cells. **P < 0.01; *P < 0.05.

The correlation between mRNA expression of the 30 hub genes and 22 immune cell types is shown in Figure 4B. Naive T cells were not considered due to lack of expression in almost all patients, prohibiting any comparisons. mRNA expression of *MMP9* and *SPP1* showed a high positive correlation with Macrophage M0 cells (Figure 4A). mRNA expression of IL1B showed a high positive correlation with activated mast cells and neutrophils. In contrast, mRNA expression of *MMP9* showed a negative correlation with resting mast cells. The correlation between the expression of other hub genes and immune cell types was low (between 0 and 0.4). Therefore, we assumed that mRNA expression level of other hub genes, other than those mentioned above, had little correlation with immune cells.

### Correlation Between Hub Genes and TMB, TME Score, Tumor Purity, Immune Score, and Stromal Score

**FIGURE 5:**
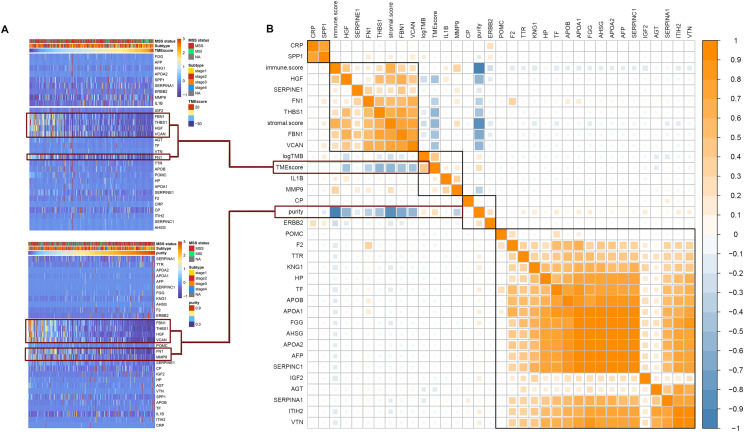
Correlation between hub genes expression and important factors. **(A)** Heat map of the hub genes expression according to the TME score and tumor purity, **(B)** correlation between hub gene expression and TMB, TME score, tumor purity, immune score, and stromal score.

Among the 30 hub genes, six genes (*FBN1, FN1, HGF, MMP9, THBS1*, and *VCAN*) were negatively correlated with tumor purity and TME score, and positively correlated with tumor immune score and stromal score (Figure 5B). Therefore, we selected these six genes as target genes. Figure 5A shows how target gene expression changes based on tumor purity and TME score. This is consistent with the results in Figure 5B, indicating that lower mRNA expression of target genes is associated with higher tumor purity and TME score, and lower immune and stromal scores.

### Correlation Between Target Gene Mutations and TMB, TME Score, Tumor Purity, Immune Score, and Stromal Score

**FIGURE 6:**
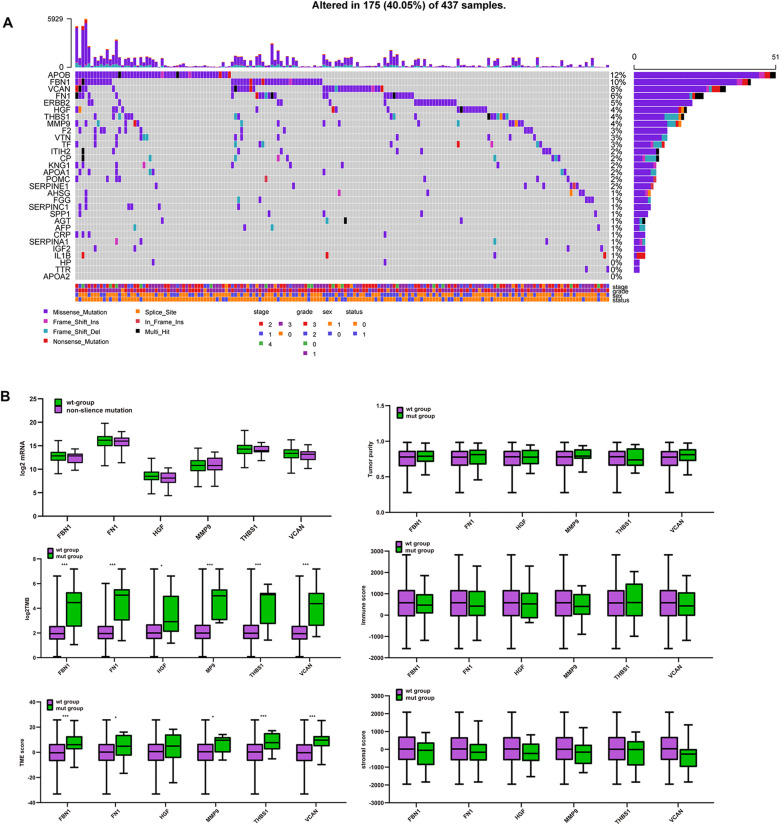
Mutation of target genes and the effect on purity, TMB and TME score. **(A)** Six hub gene mutation frequency in TCGA-STAD, **(B)** comparison of mRNA expression, tumor purity, immune score, stromal score, log2TMB, and TME score between the mutated and wild type group. ***P < 0.005; *P < 0.05.

Figure 6A shows that *APOB, FBN1, VCAN, FN1, ERBB2, HGF, THBS1*, and *MMP9* have high mutation frequencies. These genes typically harbor missense mutations. Except for *APOB* and *ERBB2*, the remaining genes were the target genes selected above, which were highly related to tumor purity, TME score, immune score, and stromal score. Figure 6B shows that there were no significant differences in mRNA expression, tumor purity, immune score, or stromal score between the mutated and wild type genes. However, the TMB and TME scores in the mutated target genes were significantly higher than those in the non-mutated genes. This suggests that GC patients with target gene mutations could displayed higher TMB and TME score.

### Correlation Between CNA of Target Genes and TMB, Tumor Purity, TME Score, Immune Score, and Stromal Score

**FIGURE 7:**
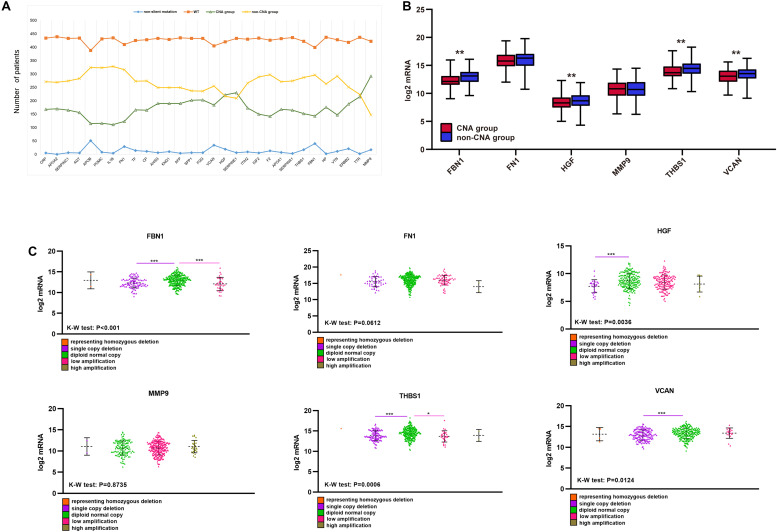
CNA of target genes and the effect on purity, TMB, and TME score. **(A)** Number of mutations and CNA for the 30 hub genes, **(B)** expression of the six target genes in the CNA and non-CNA group, **(C)** expression of the six target genes in different CNA subtype group. ***P < 0.005; **P < 0.01; *P < 0.05.

Unlike mutations, the 30 hub genes had significantly higher rates of CNA, with each gene occurring in approximately a third or more of the patients (Figure 7A). We also divided the CNA group and non-CNA group for analysis. Four target genes (*FBN1, HGF, THBS1*, and *VCAN*) had significantly lower mRNA expression in the CNA group (Figure 7B). For further analysis, we compared the mRNA expression in different subgroup (Figure 7C). When single copy deletion occurred, the expression of FBN1, HGF, THBS1, and VCAN decreased. While for FBN1 and THBS1, the expression also decreased when low amplification occurred. Maybe heterogeneity or unusual behavior of these patients affected the expression of genes. Other types of CNA seemed no impact on the expression of the genes. No matter amplification or deletion, the variation trend of genes expression reflected in Figure 7C was consistent with that in Figure 7B.

**FIGURE 8:**
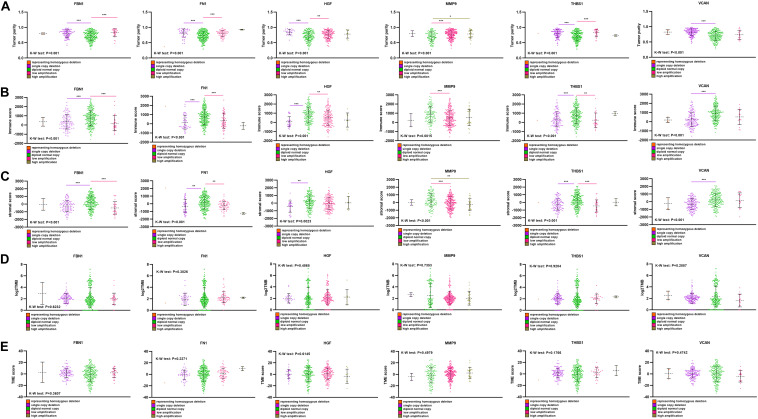
Comparison of purity, TMB, and TME score among different CNA subtype. **(A)** Tumor purity in different target gene CNA types, **(B)** immune score in different target gene CNA types, **(C)** stromal score in different target gene CNA types, **(D)** log2TMB in different target gene CNA types, **(E)** TME score in different target gene CNA types. ***P < 0.005; **P < 0.01; *P < 0.05.

Single-copy number deletions and low-fold amplifications were the major types of CNA found for the target genes. Tumor purity was higher in the CNA group than that in the non-CNA group (Figure 8A), and their stromal and immune scores were significantly lower than those in the non-CNA group (Figures 8B,C). A similar trend was found for all six target genes. There was no significant difference in TMB or TME score between the CNA and non-CNA groups (Figures 8D,E). This suggests that the CNA of target genes may significantly affected the purity of the tumor.

### Correlation Between Target Genes and Clinical Information

**FIGURE 9:**
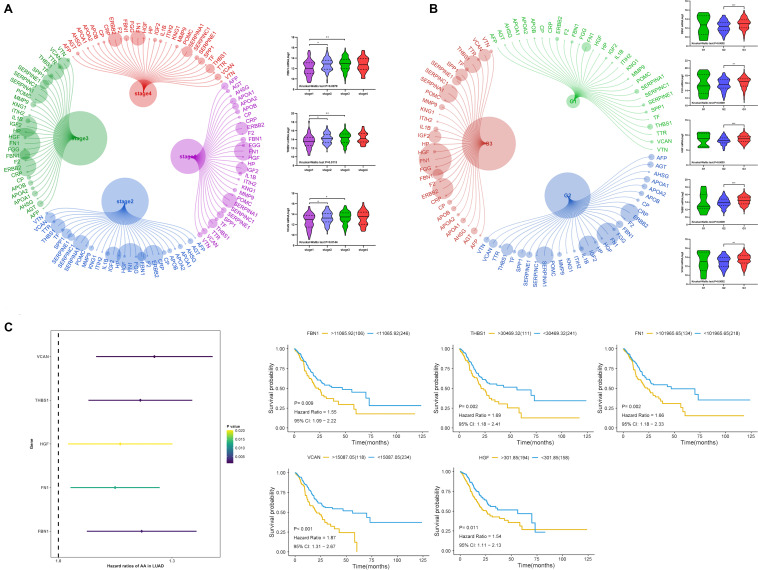
Expression of target genes and clinical characteristics. **(A)** *R*elationship between target genes and clinical stage, **(B)** relationship between target genes and clinical grade, **(C)** univariate cox analysis and Kaplan-Meier (KM) of target genes. ***P < 0.005; **P < 0.01; *P < 0.05.

We analyzed the relationship between target gene mRNA expression level and clinical stage and grade. The expression of *FBN1, THBS1*, and *VCAN* had a significant relationship with stage (Figure 9A), and each had reduced expression in stage1. Regarding grade, all target genes except *MMP9* had significant relationships with grade, especially between grade 2 and 3 (Figure 9B). This indicated that lower expression of target genes was associated with earlier stages and lower grades. Regarding survival (Figure 9C), all target genes except *MMP9* were found to be prognostic factors by univariate cox analysis; lower expression indicated better prognosis.

As shown in Table 1, most patients in the *FN1* mutation group were female, and those with *THBS1* mutations had a relatively earlier clinical stage. Other gene mutations were not clinically relevant. Patients with *VCAN* mutations appeared to have a lower grade compared to patients without *VCAN* mutations.

**TABLE 1:** Mutation of target genes and clinical characteristics.

Characteristics	FBN1			FN1			HGF		
	w	m	P	w	m	P	w	m	P
T1	22	1	0.267	21	2	0.360	21	2	0.069
T2	86	6		85	7		87	5	
T3	183	15		190	8		195	3	
T4	102	15		107	10		109	8	
T1+2	108	7	0.261	106	9	0.424	108	7	0.234
T3+4	285	30		297	18		304	11	
N0	121	12	0.677	123	10	0.582	125	8	0.769
N1	109	8		109	8		112	5	
N2	76	9		78	7		82	3	
N3	77	10		84	3		84	3	
N0+1	230	20	0.288	232	18	0.574	237	13	0.405
N2+3	153	19		162	10		166	6	
M0	353	34	0.404	363	24	0.668	370	17	0.848
M1	26	4		27	3		29	1	
Stage1	55	4	0.563	53	6	0.305	53	6	0.109
Stage2	127	9		131	5		132	4	
Stage3	164	19		171	12		177	6	
Stage4	39	5		40	4		42	2	
Stage1+2	182	13	0.157	184	11	0.556	185	10	0.416
Stage3+4	203	24		211	16		219	8	
G1	11	1	0.524	12	0	0.641	11	1	0.650
G2	140	18		147	11		153	5	
G3	239	21		243	17		250	10	
Female	142	16	0.580	141	17	0.009*	151	7	0.937
Male	257	24		269	12		269	12	

**Characteristics**	**MMP9**			**THBS1**			**VCAN**		
	**w**	**m**	**P**	**w**	**m**	**P**	**w**	**m**	**P**

T1	22	1	0.644	21	2	0.573	20	3	0.421
T2	87	5		88	4		83	9	
T3	193	5		190	8		187	11	
T4	112	5		114	3		108	9	
T1+2	109	6	0.322	109	6	0.416	103	12	0.153
T3+4	305	10		304	11		295	20	
N0	127	6	0.573	125	8	0.242	119	14	0.376
N1	112	5		111	6		108	9	
N2	82	3		84	1		80	5	
N3	86	1		85	2		83	4	
N0+1	239	11	0.258	236	14	0.084	227	23	0.130
N2+3	168	4		169	3		163	9	
M0	370	17	0.624	371	16	0.618	356	31	0.930
M1	30	0		30	0		28	2	
Stage1	57	2	0.445	55	4	0.229	54	5	0.327
Stage2	129	7		128	8		122	14	
Stage3	177	6		179	4		174	9	
Stage4	44	0		43	1		41	3	
Stage1+2	186	9	0.275	183	12	0.040^∗^	176	19	0.118
Stage3+4	221	6		222	5		215	12	
G1	11	1	0.228	12	0	0.161	9	3	0.023^∗^
G2	149	9		155	3		142	16	
G3	253	7		246	14		245	15	
Female	150	8	0.332	150	8	0.332	141	17	0.076
Male	272	9		272	9		264	17	

* Indicates p < 0.05; “w” meant wild type group; “m” meant mutated group.

Besides, we also compared the clinical characteristics in CNA subgroup (Supplementary Table S3). However, the small sample size in some group made the comparison less accurate, so we divided the patients into CNA and non-CNA group. As shown in Table 2, the number of male patients with *MMP9* CNA was higher than the number of female patients, and patients with *MMP9* CNA showed a lower grade. In addition, patients with *THBS1* CNA or *VCAN* CNA also showed lower grades.

**TABLE 2:** CNA of target genes and clinical characteristics.

Characteristics	FBN1			FN1			HGF		
	n	c	P	n	c	P	n	c	P
T1	15	4	0.076	11	8	0.065	8	11	0.321
T2	49	31		63	17		41	39	
T3	104	63		109	58		77	90	
T4	75	25		75	25		57	43	
T1+2	64	35	0.667	74	25	0.277	49	50	0.906
T3+4	179	88		184	83		134	133	
N0	81	31	0.208	76	36	0.540	54	58	0.253
N1	65	32		73	24		56	41	
N2	43	32		54	21		32	43	
N3	49	25		49	25		37	37	
N0+1	146	63	0.109	149	60	0.658	110	99	0.238
N2+3	92	57		103	46		69	80	
M0	222	107	0.535	233	96	0.765	161	168	0.286
M1	16	10		17	8		15	10	
Stage1	35	18	0.350	36	17	0.851	24	29	0.702
Stage2	80	30		75	35		55	55	
Stage3	93	57		109	41		75	75	
Stage4	25	13		27	11		22	16	
Stage1+2	115	48	0.124	111	52	0.385	79	84	0.559
Stage3+4	118	70		136	52		97	91	
G1	7	3	0.256	8	2	0.162	4	6	0.306
G2	84	53		89	48		61	76	
G3	152	66		161	57		114	104	
Female	88	46	0.825	89	45	0.158	74	60	0.096
Male	161	79		176	64		111	129	

**Characteristics**	**MMP9**			**THBS1**			**VCAN**		
	**n**	**c**	**P**	**n**	**c**	**P**	**n**	**c**	**P**

T1	5	14	0.620	13	6	0.180	10	9	0.212
T2	25	55		95	32		47	33	
T3	50	117		105	62		87	80	
T4	37	63		70	30		65	35	
T1+2	30	69	0.678	108	38	0.078	57	42	0.912
T3+4	87	180		175	92		152	115	
N0	41	71	0.433	78	34	0.190	67	45	0.473
N1	27	70		65	32		50	47	
N2	21	54		41	34		41	34	
N3	26	48		47	27		46	28	
N0+1	68	141	0.83	143	66	0.068	117	92	0.650
N2+3	47	102		88	61		87	62	
M0	105	224	0.673	214	115	0.916	185	144	0.450
M1	9	16		16	9		16	9	
Stage1	16	36	0.663	34	19	0.336	31	22	0.665
Stage2	39	71		79	32		60	50	
Stage3	45	109		91	59		84	66	
Stage4	14	24		23	15		25	13	
Stage1+2	55	107	0.518	113	51	0.106	91	72	0.685
Stage3+4	59	133		114	74		109	79	
G1	1	10	0*	7	3	0.023*	7	3	0*
G2	31	106		76	62		58	79	
G3	84	134		151	67		141	77	
Female	54	80	0.009*	87	47	0.883	81	53	0.272
Male	65	175		154	86		131	109	

* Indicates p < 0.05; “n” meant non-CNA group; “c” meant CNA group.

**FIGURE 10:**
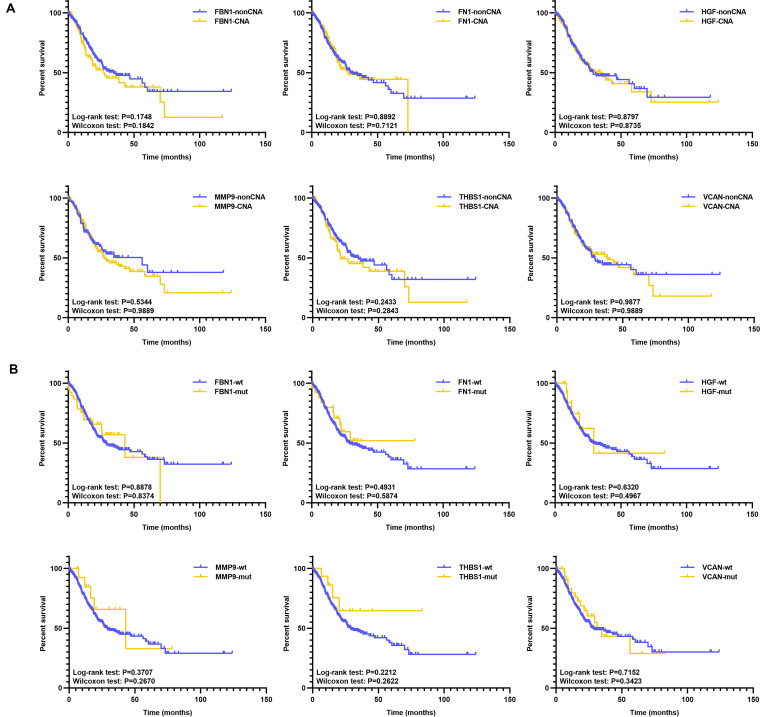
KM of target genes with mutation or CNA. **(A)** KM survival between the CNA and non-CNA group of target genes, **(B)** KM survival between the mutated and wild type group of target genes.

Additionally, we analyzed the prognosis with respect to target genes in the mutant and wild type groups, and CNA and non-CNA group; however, there were no significant differences (Figure 10 and Supplementary Table S3). This indicates that the mutation or CNA of target genes is associated with an earlier stage or grade, especially for *THBS1, VCAN, FBN1*, and *MMP9*.

### Target Genes and 10 Classic Oncogenic Signaling Pathways

Sanchez-Vega et al. (2018) showed that 10 oncogenic signaling pathways are altered in the TCGA pan-cancer atlas. The pathways were considered altered if any key pathway gene was changed (Sanchez-Vega et al., 2018). Here we cited the data of signaling pathways and gene alteration in the article, and explored the relationship between our target genes and these pathways. Indeed, the classic oncogenic pathways were found to be altered in different groups with respect to the mutation and CNA of our target genes.

**FIGURE 11:**
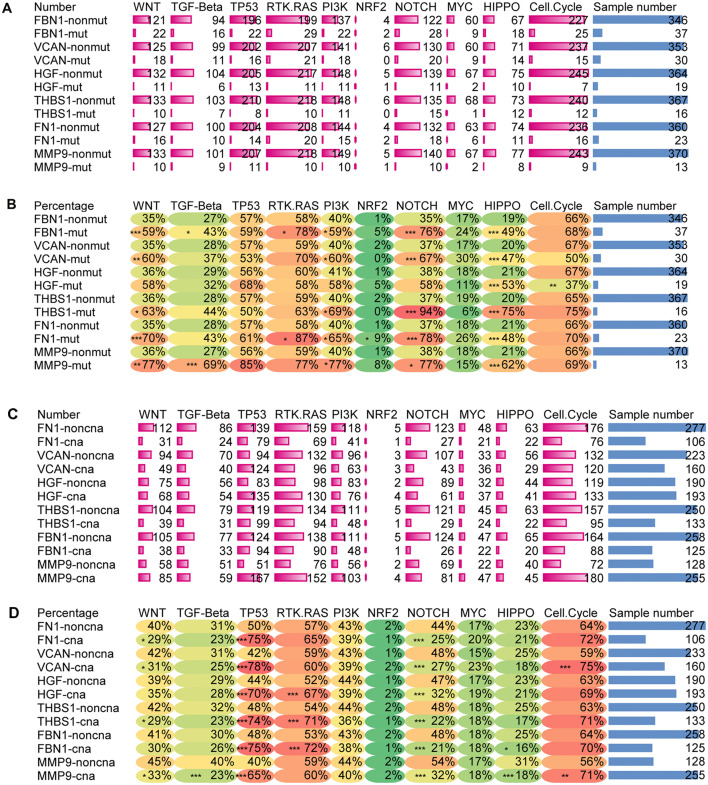
Alteration ratio of 10 signaling pathway in mutation and CNA groups. **(A)** Number of changes in each pathway for the mutated and wild type groups of target genes, **(B)** percentage of change numbers in each pathway for the mutated and wild type groups of target genes, **(C)** number of changes in each pathway for the CNA and non-CNA groups of target genes, **(D)** percentage of change numbers in each pathway for the CNA and non-CNA groups of target genes. ***P < 0.005; **P < 0.01; *P < 0.05.

Figures 11A,B shows that the mutation of target genes seemed to have no effect on the TP53 or MYC pathway. Mutations in all target genes displayed higher alterations in the HIPPO pathway. With exception of *HGF*, the mutation of target genes also showed higher alterations in the WNT, PI3K, and NOTCH pathways. In addition, mutation of *FBN1* and *MMP9* reflected higher alterations in the TGF pathway. Mutation of *FBN1* and *FN1* showed increased alterations of the RTK-RAS pathway. Mutation of *FN1* showed increased alteration in NRF2 pathway. For *HGF* mutation, other than the HIPPO pathway, only the Cell cycle pathway had reduced alteration.

Figures 11C,D shows that when CNA occurred in any of the target genes, the altered ratio of the TP53 pathway significantly increased while the altered ratio of the NOTCH pathway significantly decreased. In addition, alteration of the RAS pathway seemed to increase with the CNA of *HGF, THBS1*, and *FBN1*. Alteration of Cell cycle pathway also increased with the CNA of *VCAN* and *MMP9*. The CNA of *FN1*, *THBS1*, *VCAN*, and *MMP9* reflected a decreased alteration of the WNT pathway. CNA of MMP9 displayed a decreased alteration in the TGF pathway and HIPPO pathway. CNA of *FBN1* showed a reduced alteration in the HIPPO pathway. PI3K, NFR2, and MYC signaling pathways showed no significant alterations with the CNA of target genes.

This indicated that the CNA of target genes seemed to have more influence on the WNT, TP53, and NOTCH pathways. However, the mutation of target genes may have more influence on the WNT, PI3K, NOTCH, and HIPPO pathways. Taken together, these results showed that changes in the target genes could affect multiple signaling pathways.

### Co-occurrence and Mutual Exclusivity Between Target Genes and Pathway Genes

**FIGURE 12:**
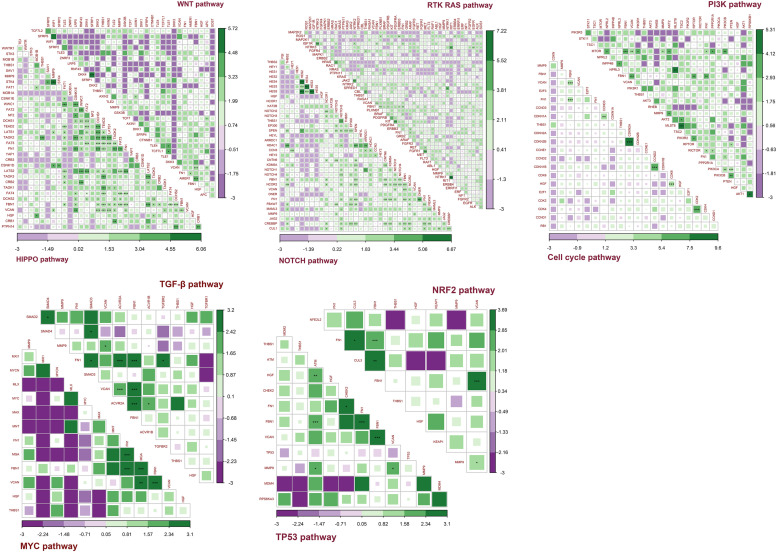
Co-occurrence and mutual exclusivity between target genes and key genes in each pathway. Green indicates co-occurrence and purple indicates mutual exclusivity; ***P < 0.005; **P < 0.01; *P < 0.05.

Figure 12 shows the co-occurrence and mutual exclusivity between target genes and 187 genes from 10 pathways. Target genes exhibited co-occurrence with the key genes in these pathways and no significant mutual exclusivity (Supplementary Table S4). In addition, *FN1* and *FBN1* showed more co-occurrence with pathway genes, particularly from the NOTCH and HIPPO pathways. The other target genes also tended to co-occur with genes in the NOTCH and HIPPO pathways. Among the target genes, *FBN1* most often co-occurred with *FN1* and *VCAN*. *MMP9* most often co-occurred with *VCAN*. *HGF* and *THBS1* have little co-occurrence with other genes (Supplementary Table S4).

Together, these results indicated that the target genes always exhibited co-occurrence in GC, especially *FBN1*, *FN1*, and *VCAN*. The target genes exhibited more co-occurrence with key genes in the NOTCH and HIPPO pathways than other pathways.

## Discussion

GC is a common malignant tumor with various factors causing high mortality rates and a low ratio of 5-year survival. Different treatment regimens have been adopted for GC patients at different stages. Even patients with the same pathological type, the outcomes of surgery and post-operative chemotherapy will be very different. Therefore, it has been urgent find new factors and targets in GC. In our study, we combined the GDSC, CCLE, and TCGA database to identify six new target genes *(FBN1, FN1, HGF, MMP9, THBS1*, and *VCAN*). A few of articles reflected that some indexes could affect the proliferation and invasion of GC by regulating HGF or MMP9 (Appleby et al., 2017; Matsumoto et al., 2017; Zhang et al., 2017; Ding et al., 2018; Wang R. et al., 2018), but the function of these six genes in GC was still not well-known, especially *FBN1, FN1*, and *VCAN* (Lee et al., 2016; Sakai et al., 2016; Wang J. et al., 2018; Jiang et al., 2019). By observing the relationships between target genes and important factors that have proven to affect the treatment and prognosis of GC, such as immune infiltration, tumor purity, TMB, TME score, and oncogenic signaling pathways, we expect that the six genes could be considered as new prognostic targets in GC.

In terms of mRNA, a high negative correlation was found between the target genes and purity and TME score. Studies have shown that as purity decreases, it becomes more challenging for drugs to penetrated into the tumor, leading to a lower recognition and elimination ratio by endogenous immune cells (Mao et al., 2018; Rhee et al., 2018). The TME score is an approach to estimate the microenvironment surrounding the tumor cells. Studies have found that higher TME scores show improved immune treatment and prognosis (Junttila and de Sauvage, 2013; Lazar et al., 2018; Zeng et al., 2019). In our study, all six genes, except *MMP9*, showed a significant negative correlation with tumor purity and TME score. Additionally, improved survival was observed in lower mRNA expression groups. Meanwhile, low mRNA expression of *FBN1, THBS1*, and *VCAN* was observed in earlier stages, and lower expression of all six genes, except *MMP9*, was observed in earlier grades. Taken together, this indicates that the lower expression of these target genes may play a role in improving tumor purity and TME to display a better prognosis of GC.

Regarding somatic mutation, significantly higher TMBs and TME scores were observed in groups with mutations in the target genes. TMB was calculated based on the number of mutations per gene. Patients with higher TMB have been found to have a better prognosis (Hellmann et al., 2018; Maleki Vareki, 2018; Wang F. et al., 2019; Kim et al., 2020). As the tumor reaches a higher TMB, it is more likely to have more neoantigens that could be recognized and killed by endogenous immune cells. Additionally, TME scores have been shown to have a positive correlation with TMB (Zeng et al., 2019). In our study, the mutation frequency of the target genes is shown in Figure 6. When the six genes have mutations, their mRNA expression level is not affected, but the TMB increased in all mutated groups. Additionally, higher TME scores were observed in all target genes except *HGF*. No significant difference was found for tumor purity, immune score, or stromal score. In addition, *THBS1* mutations were associated with an earlier stage while the *VCAN* mutations were associated with a lower grade. Taken together, these results indicate that the mutation of these target genes may affect the clinical stage and grade, and improve the prognosis or treatment by affecting the TMB and TME score.

CNA primarily affects tumor purity. Five CNA types, including homozygous deletion, single copy deletion, normal copy, low amplification, and high amplification, were observed, with single copy deletion and low amplification being the most common. CNA of each target gene reflected higher tumor purity and lower immune and stromal scores. No significant difference was observed in TMB or TME score. In addition, the CNA of four target genes (*FBN1, HGF, THBS1*, and *VCAN*) led to lower mRNA expression of the gene compared to a normal group. Lower expression of target genes was associated with better prognosis, as shown in Figure 9. Therefore, we believe that the CNA of target genes may significantly reduce their mRNA expression and improve the purity of the tumor to affect the prognosis of GC.

To explore the possible pathways affected by the six target genes, we investigated the relationship between the target genes and 10 classic oncogenic signaling pathways that have been proven to play important roles in the occurrence and development of cancer (Joshi-Tope et al., 2005; Ciriello et al., 2013; Bahceci et al., 2017; Sanchez-Vega et al., 2018). The co-occurrence and mutual exclusivity between the key genes of each pathway and our target genes were calculated (Tyner et al., 2018). The target genes are related to multiple pathways. Mutations of the target genes can be accompanied by alterations in multiple pathways, including mutations, methylation, CNA, etc. In addition, the target genes tended to co-occur with key genes in the NOTCH and HIPPO pathways. Some genes that targets co-occurred with, such as *BRAF, PDGFRB, APC, IGF1R*, and *MTOR*, have been targeted by drugs to treat tumor progression (Wang et al., 2014; Kavuri et al., 2015; Long et al., 2015; Smyth et al., 2016; David et al., 2017; Zhang et al., 2018; Sullivan et al., 2019). In the clinical treatment of patients with alterations in the target genes, we can choose to study the classic pathway where these relevant pleiotropic genes are located to then apply relevant drugs for clinical treatment. This will further clarify our research and therapeutic directions, and enhance treatment.

In summary, *FBN1, FN1, HGF, MMP9, THBS1*, and *VCAN* can be used as new target genes to observe the prognosis of gastric cancer. The lower the expression, the better the prognosis. The mutation of target genes may affect the TMB and TME score of the tumor, while their CNA may make an impact on the purity of the tumor. By exploring the relationship between target genes and hub genes in oncogenic signaling pathways that they co-occur with, such as *BRAF, PIK3CA, APC, MTOR*, etc., that have been proven in targeted therapy, we can choose more suitably potential research mechanisms to improve the prognosis of GC. We will further collect clinical samples and study the mechanisms in detail through *in vitro* and *in vivo* experiments.

## Data Availability Statement

Publicly available datasets were analyzed in this study. This data can be found here: https://portals.broadinstitute.org/ccle; https://www.cancerrxgene.org/; https://www.cancer.gov/; https://xena.ucsc.edu/.

## Author Contributions

YL and L-PL were the guarantors and designed the study. YL, J-SW, and H-CW participated in the acquisition, analysis, and interpretation of the data, and drafted the initial manuscript. TZ participated in the statistics analysis. J-SW and L-PL completed the review and editing. All authors contributed to the article and approved the submitted version.

## Conflict of Interest

The authors declare that the research was conducted in the absence of any commercial or financial relationships that could be construed as a potential conflict of interest.

## Footnotes

1 https://portals.broadinstitute.org/ccle

2 https://www.cancerrxgene.org/

3 https://www.cancer.gov/

4 http://gepia2.cancer-pku.cn/#index

5 https://xena.ucsc.edu/

6 https://david.ncifcrf.gov/

7 https://string-db.org/

8 https://cibersort.stanford.edu/

## Abbreviations

5-FU: 5-Fluorourac
CCLE: Cancer Cell Line Encyclopedia
CNA: Copy Number Alteration
GC: Gastric Cancer
GDSC: Genomics of Drug Sensitivity in Cancer
GO-BP: Gene Ontology Biological process
GTEx: The Genotype-Tissue Expression
KM: Kaplan-Meier
STAD: Stomach Adenocarcinoma
TCGA: The Cancer Genome Atlas
TMB: Tumor Mutation Burden
TME: Tumor Microenvironment.
